# Preliminary Clinical Case Series for Evaluation of a Novel Bi-Dimensional Archwire During Miniscrew-Supported En-Masse Retraction of the Maxillary Anterior Teeth

**DOI:** 10.3390/reports9040334

**Published:** 2026-10-02

**Authors:** Abdulmalek M. H. Almasri, Mohammad Y. Hajeer, Ahmad S. Burhan, Safwan Jaber

**Affiliations:** 1 Department of Orthodontics, Faculty of Dentistry, University of Damascus, Damascus P.O. Box 16046, Syria; abdulmalekalmasri@gmail.com (A.M.H.A.); dr.burhan-a@hotmail.com (A.S.B.); 2 Department of Oral and Maxillofacial Surgery, Faculty of Dentistry, Arab International University, Daraa P.O. Box 16046, Syria; s-jaber@aiu.edu.sy

**Keywords:** Class II malocclusion, en-masse retraction, maxillary anterior teeth, bi-dimensional archwire, torque control, miniscrew-supported anchorage, clinical case series

## Abstract

Background/Objectives: Efficient en-masse retraction of the maxillary anterior teeth requires adequate anchorage control while maintaining acceptable incisor torque. This prospective clinical case series aimed to provide an initial clinical evaluation of a novel laser-welded bi-dimensional archwire designed for miniscrew-supported en-masse retraction in adult patients with Class II Division 1 malocclusion. Methods: Six patients requiring extraction of the maxillary first premolars were treated using a custom-made stainless-steel archwire consisting of a 0.019 × 0.025-inch anterior segment and 0.016 × 0.022-inch posterior segments. Retraction was performed using nickel-titanium closed-coil springs delivering approximately 250 g of force per side. The primary outcome was the monthly rate of maxillary anterior retraction measured on three-dimensional digital models. The secondary outcome was the change in maxillary incisor torque assessed on lateral cephalometric radiographs using the bracket–archwire angle. Results: Repeated measurements showed no statistically significant systematic error, with method errors ranging from 0.14 to 0.31 mm for linear variables and from 0.72° to 1.08° for angular variables; intraclass correlation coefficients ranged from 0.91 to 0.97. The overall mean retraction rate was 0.97 ± 0.05 mm/month, and the mean retraction duration was 5.78 ± 0.79 months. The mean change in maxillary incisor torque was 7.08 ± 1.29°. Conclusions: Within the limitations of this six-participant clinical case series, the novel bi-dimensional archwire was associated with a relatively stable observed rate of anterior retraction, although a measurable change in the bracket–archwire angular relationship remained. These descriptive findings should be regarded as preliminary and hypothesis-generating and require confirmation in adequately powered controlled studies. Trial registration: ClinicalTrials.gov, NCT07664215; retrospectively registered on 24 June 2026.

## 1. Introduction

Class II division 1 malocclusion is a common orthodontic condition, typically characterized by proclined maxillary incisors, increased overjet, and a convex facial profile [1]. In moderate to severe cases, extraction of maxillary premolars is frequently indicated to create sufficient space for anterior tooth retraction, reduce dentoalveolar protrusion, and improve facial esthetics [2]. The success of such treatment protocols is highly dependent on precise biomechanical control during the space-closure phase, particularly with respect to anchorage preservation, torque expression, and the pattern of anterior tooth movement [3].

Anchorage control remains a major biomechanical challenge during en-masse retraction of the maxillary anterior segment. Inadequate anchorage may result in undesirable mesial movement of posterior teeth, inefficient utilization of extraction space, and prolonged treatment duration [4]. The introduction of temporary anchorage devices (TADs), particularly miniscrews, has significantly enhanced anchorage control during space closure [5]. Miniscrew-supported en-masse retraction allows posterior anchorage reinforcement and minimizes unwanted mesial movement of posterior teeth, thereby improving the predictability of extraction-based treatments [6,7]. However, while miniscrews effectively address anchorage demands, they do not inherently resolve biomechanical limitations related to archwire–bracket interactions and force delivery to the anterior teeth [8,9]. The configuration and mechanical properties of the archwire play a decisive role in determining frictional resistance, force transmission, and torque control during sliding mechanics [3,10]. Conventionally, en-masse retraction is performed using a single continuous rectangular stainless-steel archwire, most commonly a 0.019 × 0.025-inch wire engaged in a 0.022-inch bracket system [2]. Although this configuration provides sufficient rigidity for space closure, several clinical and experimental studies have reported persistent challenges related to incisor torque loss, anterior tipping, and archwire deformation under retraction forces [11,12].

To overcome these limitations, alternative biomechanical strategies have been proposed to enhance torque control while reducing friction [13]. The bi-dimensional archwire technique employs differential wire dimensions between the anterior and posterior segments to optimize moment-to-force ratios and improve three-dimensional control during space closure [14]. Biomechanical and finite-element investigations have demonstrated that incorporating differential stiffness within the archwire system can reduce wire deflection, limit occlusal plane rotation, and improve anterior segment control during miniscrew-supported retraction [11].

Building on the same biomechanical rationale, the concept of a bi-dimensional archwire—characterized by a stiffer anterior section combined with a more flexible posterior segment—has emerged as a clinically practical alternative [15]. By maintaining a full-size rectangular wire anteriorly to preserve incisor torque and transitioning posteriorly to a smaller cross-section to reduce friction and binding, bi-dimensional archwire designs aim to balance torque preservation with enhanced retraction efficiency [16]. Preliminary clinical and experimental evidence suggests that differential-section archwire systems may offer favorable biomechanical characteristics for sliding mechanics and anterior tooth control [15].

Recent randomized clinical trials evaluating miniscrew-assisted en-masse retraction have emphasized the influence of force-system optimization on retraction rate, dental and skeletal responses, and patient-reported outcomes [6,17,18,19,20]. In parallel, systematic reviews assessing friction-reduced and alternative retraction mechanics have highlighted the ongoing need for biomechanically efficient approaches that minimize friction while maintaining precise torque control of the anterior teeth [21]. Despite increasing interest in segmented and bi-dimensional archwire systems [22], clinical evidence regarding their performance during miniscrew-supported maxillary anterior en-masse retraction remains limited, particularly concerning retraction rate and incisor torque control.

Despite the biomechanical rationale supporting differential-section archwires, clinical evidence regarding this specific laser-welded configuration remains unavailable. An initial prospective clinical evaluation was therefore undertaken to obtain descriptive clinical data before the design of a larger comparative investigation. Accordingly, this prospective clinical case series aimed to describe the observed rate of maxillary anterior en-masse retraction and the change in the bracket–archwire angular relationship, which is used as a surrogate measure of maxillary incisor torque during miniscrew-supported retraction with the novel bi-dimensional archwire.

## 2. Materials and Methods

### 2.1. Study Design and Settings

This was a prospective clinical case series evaluating a bi-dimensional archwire system during miniscrew-supported en-masse retraction of the maxillary anterior teeth. The study was conducted at the Department of Orthodontics, Faculty of Dentistry, Damascus University, between July 2025 and April 2026. This investigation was conducted as part of a broader clinical research project evaluating biomechanical strategies to improve torque control during extraction-based orthodontic treatment. Ethical approval for the overall research protocol was obtained from the Research Ethics Committee of the Faculty of Dentistry at Damascus University (DN-300625-479). The clinical case series was retrospectively registered at the ClinicalTrials.gov database (NCT07664215) on the 24th of June, 2026. This clinical case series is reported in accordance with the Transparent Reporting of Evaluations with Nonrandomized Designs (TREND) statement, where applicable. No separate prospectively prepared study protocol was available before participant enrollment, and no study protocol was deposited in a public repository. No blinding was applied in this single-arm study.

### 2.2. Study Population

Participants were recruited from patients seeking orthodontic treatment at the Department of Orthodontics, Faculty of Dentistry, Damascus University. Eligibility was determined according to predefined inclusion and exclusion criteria. Eligible participants were adults aged 18–28 years with skeletal Class II malocclusion (ANB angle 5–7°), Class II Division 1 dental malocclusion according to Angle classification, an average or vertical facial growth pattern, a treatment plan requiring extraction of the maxillary first premolars, a need for maxillary anterior en-masse retraction, and the presence of all permanent maxillary teeth except the third molars. Patients were excluded if they had undergone previous orthodontic treatment, had craniofacial anomalies, systemic diseases affecting bone metabolism, active periodontal disease, poor oral hygiene, missing maxillary permanent teeth, or were using medications known to influence bone metabolism. After confirming eligibility, the study objectives and procedures were explained to all participants, and written informed consent was obtained before enrollment. As this investigation was conducted as a descriptive clinical case series, no formal hypothesis-based sample-size estimation was performed. Of 18 patients assessed for eligibility, 10 were excluded and 2 declined participation; the remaining 6 eligible and consenting participants constituted the case series.

### 2.3. Orthodontic Treatment Protocol

All patients were treated using fixed orthodontic appliances following a standardized treatment protocol. After extraction of the maxillary first premolars, skeletal anchorage was established using self-drilling orthodontic miniscrews (1.6 × 8 mm; 3S Screw, HUBIT Orthodontics, Gyeonggi, South Korea). The miniscrews were inserted bilaterally between the roots of the maxillary second premolars and first molars under local anesthesia. Pre-adjusted edgewise appliances with MBT prescription brackets (Pinnacle™, Ortho Technology, West Columbia, SC, USA) with a 0.022-inch slot system were bonded to the maxillary and mandibular teeth.

### 2.4. Leveling and Alignment Phase

During the leveling and alignment phase, a standardized sequence of nickel-titanium archwires was used and replaced at approximately three-week intervals as follows: 0.014-inch, 0.016-inch, 0.016 × 0.022-inch, 0.017 × 0.025-inch, and 0.019 × 0.025-inch. After completion of leveling and alignment, the working bi-dimensional stainless-steel archwire was inserted, consisting of a 0.019 × 0.025-inch anterior segment and a 0.016 × 0.022-inch posterior segment. Crimpable hooks were then placed distal to the maxillary lateral incisors to facilitate the application of retraction forces.

### 2.5. Design of the Novel Bi-Dimensional Orthodontic Archwire

The bi-dimensional archwire evaluated in this study was designed by the investigators and custom fabricated specifically for miniscrew-supported en-masse retraction of the maxillary anterior teeth. It was not a commercially available prefabricated bi-dimensional archwire. Commercially available stainless-steel orthodontic archwires (Ortho Technology, West Columbia, SC, USA) were used as the source material.

The anterior segment was fabricated from a 0.019 × 0.025-inch stainless-steel archwire and extended from approximately 2–2.5 mm distal to the canine bracket on one side to a corresponding point 2–2.5 mm distal to the contralateral canine bracket. The posterior segments were fabricated from 0.016 × 0.022-inch stainless-steel archwires. The design was intended to provide greater anterior stiffness and bracket-slot engagement while allowing a smaller posterior cross-section during sliding mechanics.

**Figure 1:**
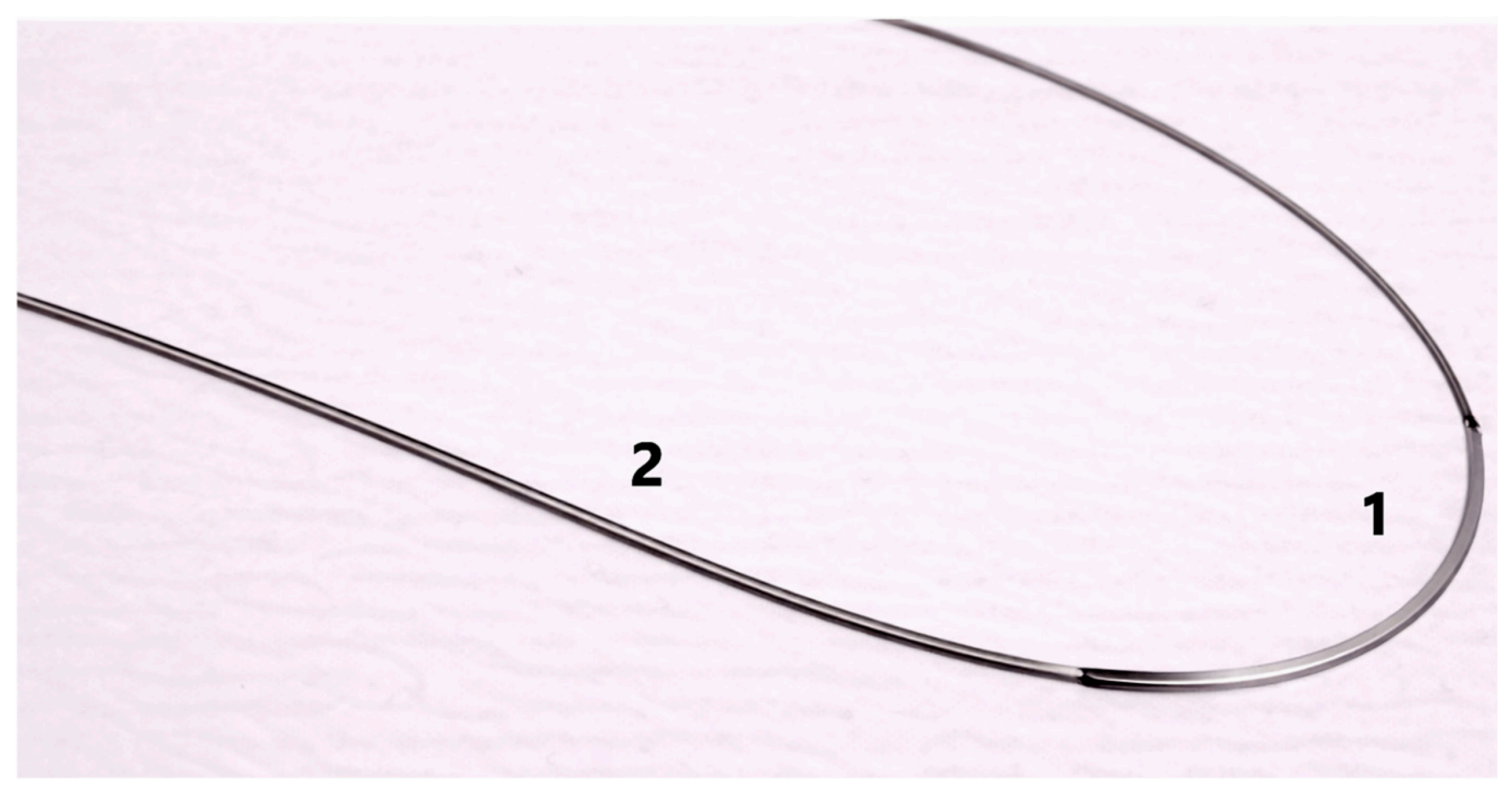
The novel bi-dimensional archwire used in this study. (1) Anterior stainless-steel segment (0.019 × 0.025-inch); (2) posterior stainless-steel segment (0.016 × 0.022-inch).

The two posterior segments were joined to the anterior segment by neodymium-doped yttrium aluminum garnet (Nd:YAG) laser welding in an external orthodontic laboratory, producing a single continuous archwire (Figure 1). Following fabrication, each archwire was visually inspected before clinical use to verify the continuity and integrity of the welded junctions and to confirm preservation of the intended arch form. Minor finishing adjustments were performed clinically when required before insertion.

The parent wires were commercially manufactured stainless-steel orthodontic archwires intended for intraoral clinical use. However, no separate mechanical characterization or independent biocompatibility testing of the laser-welded bi-dimensional configuration was performed before the present preliminary clinical investigation. The archwire design is not commercially marketed, is not subject to a patent or patent application, and the investigators have no proprietary or financial interest in the device.

### 2.6. En-Masse Retraction Mechanics

**Figure 2:**
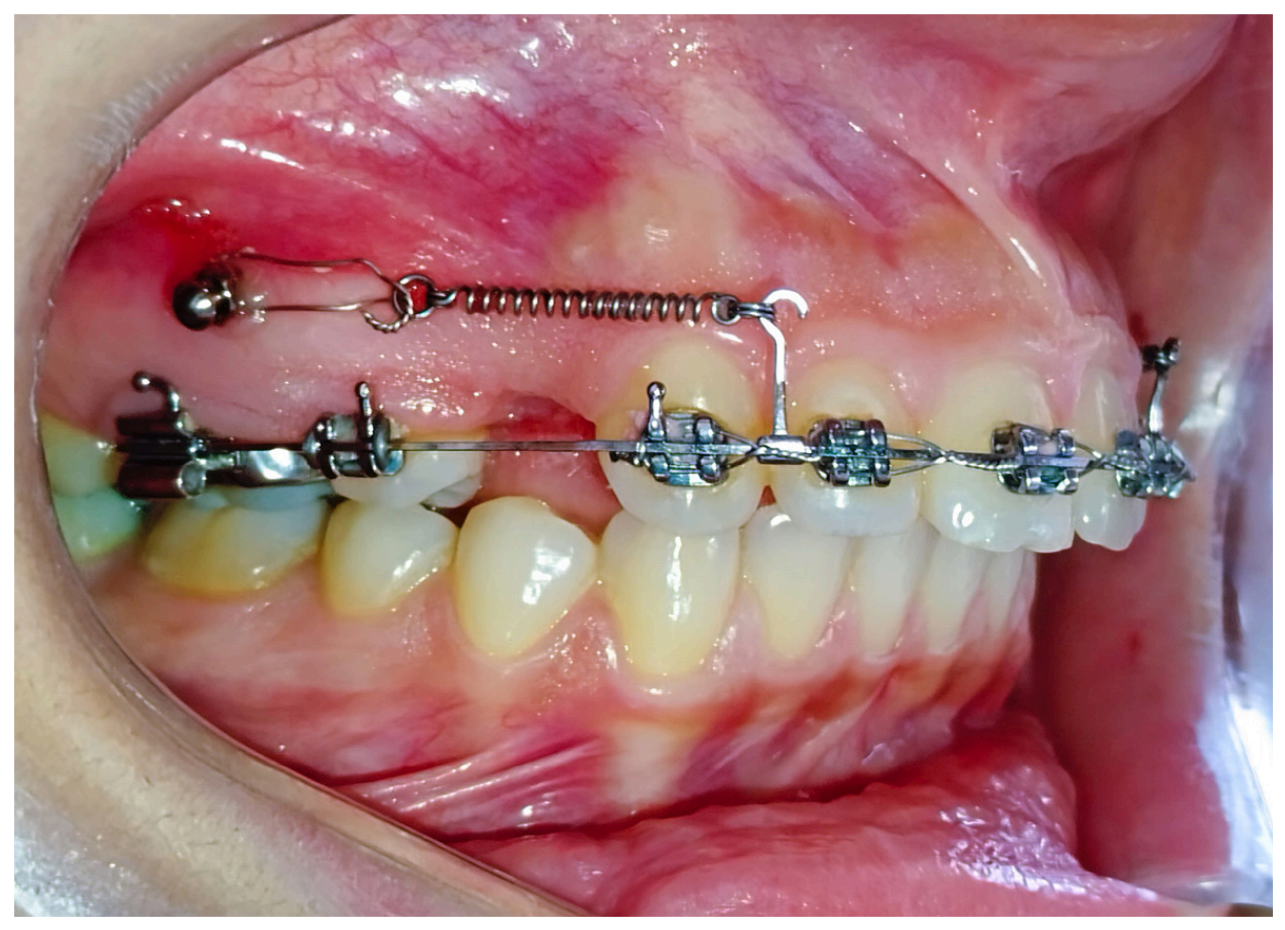
Intraoral photograph showing miniscrew-supported en-masse retraction of the maxillary anterior teeth using the bi-dimensional archwire and a nickel-titanium closed-coil spring.

After placement of the working bi-dimensional archwire, miniscrew-supported en-masse retraction of the maxillary anterior teeth was initiated. Retraction force was delivered using nickel-titanium closed-coil springs extending from crimpable hooks positioned distal to the maxillary lateral incisors to the miniscrews (Figure 2). The closed-coil spring was connected to each miniscrew by means of a stainless-steel ligature wire, the length of which was adjusted to achieve the desired spring extension and force level. A force of approximately 250 g per side was applied and verified at each visit using a Correx intraoral tension gauge (Haag-Streit, Köniz, Switzerland). During follow-up visits, the ligature wire length was readjusted as needed to maintain the same force magnitude throughout the space-closure phase. Patients were reviewed at approximately 2-week intervals to monitor retraction progress and force consistency.

### 2.7. Outcome Measures

**Figure 3:**
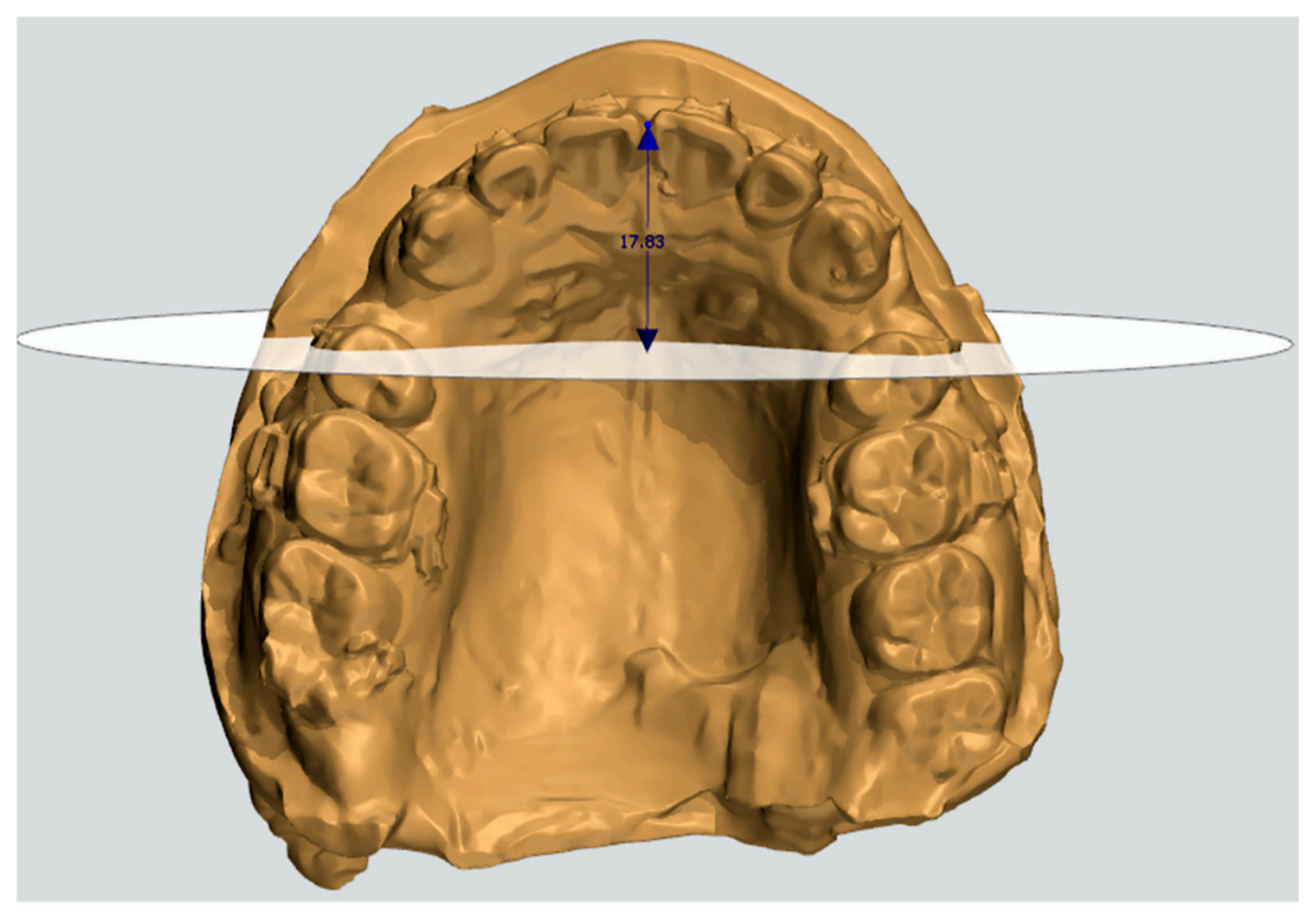
Measurement of maxillary anterior retraction on the digital dental model using the distance between the incisal edge of the maxillary central incisor and the medial point of the third palatal rugae.

The primary outcome measure was the monthly rate of maxillary anterior en-masse retraction achieved with the bi-dimensional archwire system. Anterior tooth movement was assessed on three-dimensional digital dental models obtained at predefined time points during the retraction phase. Alginate impressions were taken after the leveling and alignment phase (T0) and subsequently at monthly intervals until the completion of the retraction phase, defined by the establishment of a Class I canine relationship. All casts were digitized using a laboratory scanner with an AutoScan DS-EX Pro (SHINING 3D, Hangzhou, Zhejiang, China), and measurements were performed with 3Shape Ortho Analyzer software (3Shape, Copenhagen, Denmark). The amount of anterior retraction was determined using the distance between the incisal edge of the maxillary central incisor and the medial point of the third palatal rugae. This landmark was selected as an intraoral reference because it could be consistently identified across serial digital models and was located away from the actively retracted anterior segment (Figure 3).

The average monthly retraction rate was calculated in millimeters per month by dividing the total amount of anterior retraction by the total duration of the retraction phase.

**Figure 4:**
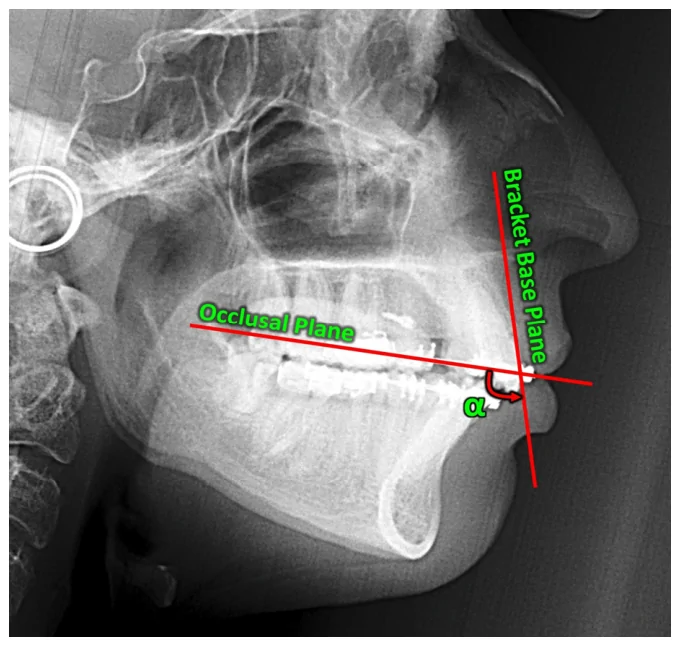
Measurement of maxillary incisor torque on the lateral cephalometric radiograph using the angle formed between a tangent drawn along the base of the maxillary central incisor bracket and another tangent parallel to the working archwire.

The secondary outcome measure was the change in maxillary incisor torque during the retraction phase. Standardized lateral cephalometric radiographs were taken at the completion of leveling and alignment (T0) and again after completion of en-masse retraction (T1). Torque measurements were carried out using ImageJ software, version 1.54 (National Institutes of Health, Bethesda, MD, USA). To determine maxillary incisor torque, a tangent was drawn along the base of the maxillary central incisor bracket, and another tangent was drawn parallel to and superimposed on the working archwire. The obtuse (α) angle formed by the intersection of these two tangents was measured, and the actual torque value was obtained by subtracting 90° from this angle (Figure 4). This method was based on the protocol described in previous studies [23,24].

### 2.8. Measurement Reliability Assessment

Measurement reliability was assessed by repeating all measurements on the lateral cephalometric radiographs and digital models of the entire study sample after a 2-week interval by the same examiner. The differences between the first and second measurements were evaluated to confirm the consistency of the measurement procedure.

### 2.9. Statistical Analysis

Statistical analyses were performed using IBM SPSS Statistics for Windows, version 22.0 (IBM Corp., Armonk, NY, USA). Descriptive statistics were used to summarize all study variables. For the primary outcome, the average monthly rate of maxillary anterior en-masse retraction was calculated in millimeters per month by dividing the total retraction distance by the total duration of the retraction phase. For the secondary outcome, the change in maxillary incisor torque between T0 and T1 was described using mean, standard deviation, minimum, and maximum values.

## 3. Results

### 3.1. Measurement Reliability

Prior to the main outcome analysis, intra-examiner measurement reliability was assessed by repeating all measurements on the lateral cephalometric radiographs and digital models of the entire study sample after a 2-week interval. The repeated measurements demonstrated acceptable reliability, with no statistically significant systematic differences between the first and second measurements (*p* > 0.05). The method error ranged from 0.14 to 0.31 mm for the linear measurements and from 0.72° to 1.08° for the angular measurements. Furthermore, the intraclass correlation coefficient values ranged from 0.91 to 0.97, indicating good measurement consistency.

### 3.2. Participant Characteristics

**Figure 5:**
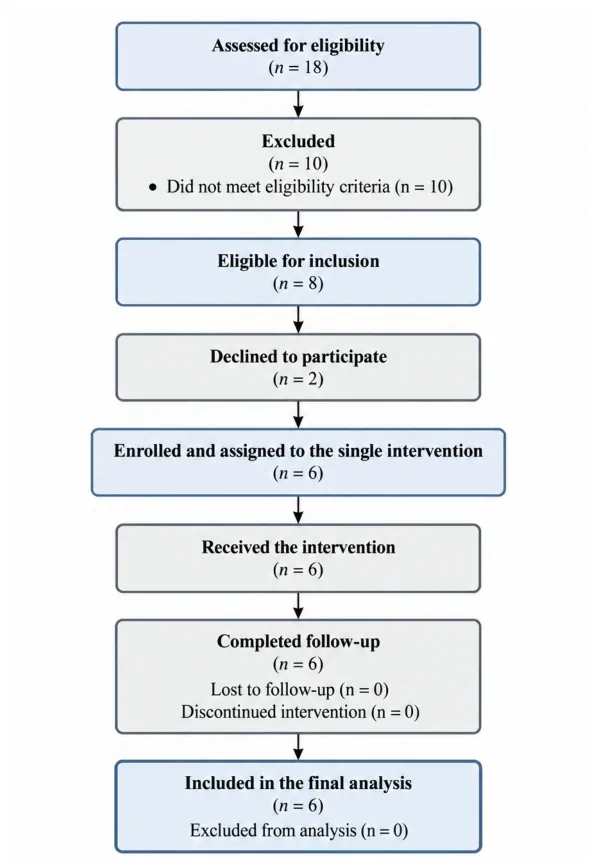
Flow diagram of participant selection for the study.

Eighteen patients were assessed for eligibility. Ten patients were excluded because they did not meet the eligibility criteria, and two eligible patients declined to participate. Six participants were enrolled and received the intervention. All six participants completed the study and were included in the final analysis, with no loss to follow-up (Figure 5). The sample consisted of two males and four females, with a mean age of 20.19 ± 1.22 years. At the beginning of the retraction phase, the mean pre-retraction extraction space was 6.41 ± 0.42 mm, indicating a relatively homogeneous sample with regard to the amount of space available for maxillary anterior en-masse retraction. These baseline characteristics are presented in Table 1.

### 3.3. Maxillary Anterior Retraction Rate and Duration

The rate of maxillary anterior tooth retraction remained relatively stable throughout the different observation intervals. During the first month of retraction (T0–T1), the mean retraction rate was 0.94 ± 0.07 mm/month, with a median of 0.94 mm/month and a range from 0.84 to 1.05 mm/month. In the second month (T1–T2), the mean rate increased slightly to 0.97 ± 0.08 mm/month, while in the third month (T2–T3), it reached 0.99 ± 0.07 mm/month. The highest mean monthly retraction rate was observed during the fourth interval (T3–T4), amounting to 1.01 ± 0.09 mm/month, after which the rate remained nearly unchanged during the fifth interval (T4–T5) at 1.03 ± 0.08 mm/month. Overall, these findings suggest that the retraction rate was maintained at a relatively consistent level throughout the space-closure phase. When the entire retraction period was considered, the overall mean rate of maxillary anterior tooth retraction from T0 to TF was 0.97 ± 0.05 mm/month, with a median value of 0.97 mm/month and values ranging between 0.90 and 1.04 mm/month. The mean time required to complete en-masse retraction was 5.78 ± 0.79 months, with a median of 5.70 months, a minimum of 4.80 months, and a maximum of 6.90 months. The descriptive statistics of the retraction rate and retraction time are summarized in Table 2.

### 3.4. Maxillary Incisor Torque Change

Regarding torque control, the mean change in maxillary incisor torque following completion of the retraction phase was 7.08 ± 1.29°. The median torque change was 7.15°, with observed values ranging from 5.29° to 8.92°. These findings indicate that, although some degree of torque loss occurred during en-masse retraction, its magnitude remained within a relatively narrow range across the study sample.

### 3.5. Adverse Events

No serious adverse events or complications requiring discontinuation of orthodontic treatment occurred. Transient peri-miniscrew inflammation was observed during follow-up and was successfully managed with oral-hygiene instructions and antiseptic mouthrinses, without interruption of orthodontic treatment.

## 4. Discussion

The present clinical case series was undertaken to provide an initial clinical evaluation of a novel bi-dimensional archwire specifically designed for miniscrew-supported en-masse retraction of the maxillary anterior teeth. The main findings were a relatively stable overall retraction rate of 0.97 ± 0.05 mm/month, completion of the retraction phase within 5.78 ± 0.79 months, and a mean change in maxillary incisor torque of 7.08 ± 1.29°. Taken together, these findings suggest that the proposed archwire design was clinically feasible and capable of producing steady anterior retraction under skeletal anchorage, although a measurable degree of torque loss still occurred during space closure.

From a biomechanical perspective, the rationale of the present archwire design was to combine the torque-preserving advantage of a full-size anterior rectangular segment with the friction-reducing effect of smaller posterior segments during sliding mechanics. This concept is consistent with previous work on bidimensional mechanics. Li and co-workers reported that bidimensional techniques enhanced anterior torque control in extraction cases compared with conventional mechanics, supporting the principle that differential anterior–posterior engagement may improve control of the upper incisors during space closure [23]. Likewise, a finite-element study by Hamanaka et al. specifically examined dual-section archwires for en-masse retraction with skeletal anchorage, further reinforcing the biomechanical plausibility of this approach [11]. In the present study, the torque change remained relatively consistent across patients, but it was not negligible, which indicates that the novel wire may improve the mechanics of retraction without completely eliminating torque loss.

With regard to retraction efficiency, the overall rate observed in this study was clinically favorable and remained relatively stable over the monthly observation intervals. This pattern is in agreement with the prospective clinical study of Gnaneswar and Sridhar, in which dual-dimensional wires showed greater space closure than conventional rectangular wires over a four-month period, with no significant difference in anchorage loss [14]. The mean space closure reported in that study for the dual-dimensional wire group was 3.98 mm over four months, which corresponds to a monthly rate very close to the value observed in the present study. Such agreement is noteworthy because it suggests that modifying the working archwire itself, rather than only the bracket prescription, may offer a practical way to improve the efficiency of en-masse retraction while preserving acceptable control.

This interpretation is also supported by recent interventional studies on miniscrew-supported en-masse retraction that evaluated other contemporary approaches for improving treatment efficiency under controlled clinical conditions [20,25].

The rate and duration recorded in the present study also appear compatible with the broader literature on miniscrew-supported en-masse retraction. Al-Sibaie and Hajeer reported that en-masse retraction supported by mini-implants yielded superior outcomes to conventional two-step retraction with traditional anchorage, particularly in terms of speed and anchorage preservation [6]. Similarly, clinical studies by Upadhyay and colleagues established the efficiency of mini-implants as intraoral anchorage units for retraction of the maxillary anterior segment [26]. Although the present investigation did not include a direct control group, the relatively short mean retraction time of 5.78 months suggests that the novel bi-dimensional archwire functioned effectively within a skeletal anchorage protocol and did not compromise the clinical efficiency generally expected from miniscrew-supported space closure. Direct comparisons should nevertheless be interpreted cautiously because differences in bracket prescription, archwire dimensions, force magnitude, hook height, miniscrew position, and outcome definitions can substantially influence both the rate and pattern of tooth movement.

The finding related to torque change deserves particular attention. A recent systematic review by Kuc et al. concluded that retroclination of the upper incisors during retraction remains a common finding across multiple treatment approaches, with a pooled mean change of 2.46°, and that properly performed corticotomy and en-masse retraction supported by orthodontic mini-implants seem to be among the most effective methods for torque control [13]. The torque change observed in the present study was numerically greater than the pooled estimate. However, this comparison should be interpreted with caution because the present study assessed torque through the bracket-archwire angle. In contrast, many of the studies included in that review relied primarily on cephalometric measures of incisor inclination. Even so, the present findings indicate that the novel archwire should not be viewed as a complete solution to torque loss; rather, it may represent a promising mechanical modification whose clinical value lies in balancing efficient sliding mechanics with a reasonable level of anterior control.

### Limitations

This clinical case series should be interpreted in light of several limitations. Its single-arm design, recruitment-driven sample of only six participants, and absence of a comparator group preclude conclusions regarding efficacy, superiority, or equivalence and substantially limit the generalizability of the findings. The single-center setting, narrow age range, and limited demographic diversity further restrict external validity. In addition, no separate prospectively prepared study protocol was available, limiting independent verification of prespecified methodological and outcome-related decisions. Maxillary incisor torque was assessed indirectly using the bracket–archwire angle on lateral cephalometric radiographs; therefore, this measure does not directly represent three-dimensional root position and may be influenced by bracket positioning. The mechanical properties and reproducibility of the laser-welded assembly were not independently characterized, and no dedicated biocompatibility testing of the modified configuration was performed, although the parent components were commercially available stainless-steel orthodontic wires intended for intraoral use. Finally, follow-up was limited to the active retraction phase, and clinically relevant outcomes such as anchorage loss, root resorption, post-treatment stability, and patient-reported outcomes were not assessed. Accordingly, the present findings should be regarded as preliminary and warrant confirmation in larger, controlled, and more diverse prospective studies.

## 5. Conclusions

Within the limitations of this small single-arm clinical case series, the novel laser-welded bi-dimensional archwire was associated with a relatively stable observed rate of maxillary anterior retraction. A measurable change in the bracket–archwire angular relationship was also observed during the retraction phase. These findings should be interpreted as preliminary descriptive observations rather than evidence of clinical superiority or effectiveness. Further adequately powered controlled prospective studies involving larger and more diverse samples are required to evaluate the clinical performance of this archwire configuration.

## Figures and Tables

**Table 1:** Baseline characteristics of the study participants at the beginning of the retraction phase.

Characteristic	Value
Number of patients	6
Gender (male/female)	2/4
Mean age (years) ± SD	20.19 ± 1.22
Pre-retraction extraction space (mm) ± SD	6.41 ± 0.42

SD, standard deviation; mm, millimeter.

**Table 2:** Descriptive statistics of the en-masse retraction rate and overall time.

Variable	Interval	Mean ± SD	Median	Max	Min
Upper anterior teeth retraction rate	T0–T1	0.94 ± 0.07 mm/month	0.94	1.05	0.84
	T1–T2	0.97 ± 0.08 mm/month	0.97	1.09	0.86
	T2–T3	0.99 ± 0.07 mm/month	0.99	1.10	0.89
	T3–T4	1.01 ± 0.09 mm/month	1.00	1.14	0.88
	T4–T5	1.03 ± 0.08 mm/month	1.03	1.15	0.91
	T0–TF	0.97 ± 0.05 mm/month	0.97	1.04	0.90
Retraction time	T0–TF	5.78 ± 0.79 months	5.70	6.90	4.80

SD, standard deviation; mm, millimeter; Max, maximum value; Min, minimum value.

## Data Availability

The data supporting the findings of this study are available from the corresponding author upon reasonable request.
